# Predicting mortality in paraquat poisoning through clinical findings, with a focus on pulmonary and cardiovascular system disorders

**DOI:** 10.1186/s40545-023-00635-z

**Published:** 2023-10-20

**Authors:** Preechaya Tajai, Akarawat Kornjirakasemsan

**Affiliations:** 1 Department of Forensic Medicine, Faculty of Medicine, Chiang Mai University, Chiang Mai, 50200 Thailand; 2 Pharmacy Department, Nakornping Hospital, Chiang Mai, 50180 Thailand

**Keywords:** Paraquat, Poisoning, Prediction, Prognosis, Mortality

## Abstract

**Background:**

Paraquat, one of the most widely used herbicides, poses a significant risk of mortality through self-poisoning and subsequent multiple organ failure. The primary objective aimed to identify the factors associated with death in patients poisoned by paraquat.

**Methods:**

A cross-sectional retrospective review was conducted at a tertiary referral hospital over five years. Eligible patients presented with acute paraquat toxicity between 1 January 2016 and 31 December 2020. Medical records of 148 patients were reviewed.

**Results:**

The in-hospital fatality rate was found to be 21.8%. Multivariate analysis revealed that the amount of paraquat ingested and clinical presentations, particularly pulmonary and cardiovascular system disorders, were significantly associated with mortality.

**Conclusion:**

Our study highlights that the amount of paraquat ingested, along with the presence of pulmonary and cardiovascular system disorders, can serve as prognostic indicators for mortality rates in cases of paraquat poisoning. These findings have important implications for physicians in predicting the prognosis and mortality of paraquat poisoning patients.

**Graphical Abstract:**

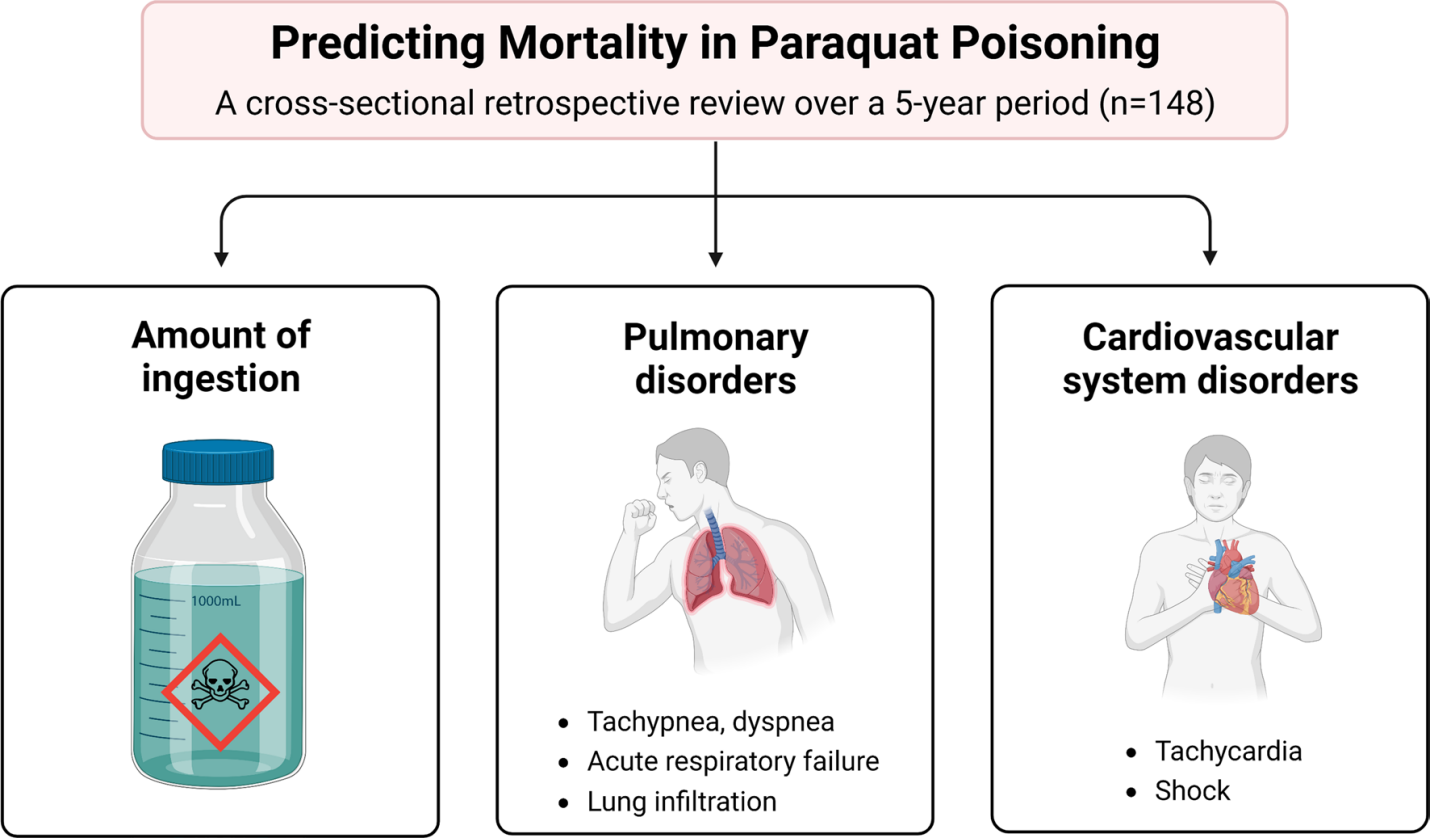

**Supplementary Information:**

The online version contains supplementary material available at 10.1186/s40545-023-00635-z.

## Introduction

Paraquat (1,1′-dimethyl, 4,4′-bipyridinium dichloride; PQ) is one of the most widely used herbicides, especially in developing countries, including Thailand [1, 2]. Self-poisoning through paraquat ingestion is a major cause of morbidity and mortality in the Asia–Pacific region [3]. In Thailand, the most common cause of acute poisoning was pesticide poisoning, accounting for 41.5% of cases. The most frequently implicated agents in these poisonings were insecticides, followed by herbicides such as glyphosate and paraquat [4]. The toxic effects of paraquat at the cellular level are believed to result from its ability to generate intracellular reactive oxygen species (ROS) through redox cycling and disrupt the mitochondrial electron transport chain [1, 2]. Moreover, an imbalance in the cellular redox state leads to significant mitochondrial damage, lipid peroxidation, and cellular toxicity [3, 5]. Paraquat can be rapidly absorbed through inhalation, ingestion, and damaged skin. Its bioavailability ranges from 0.3% to 10%. With a large volume of distribution (1.2–1.6 l/kg), it is distributed to all organs, particularly the liver, kidney, and lung. Paraquat is selectively accumulated in the lungs through an energy-dependent process involving an amino acid pump [6]. The metabolism of paraquat is limited, and it is excreted unchanged in the urine. The mean elimination half-life of paraquat is 84 h [3, 7].

Several studies have reported paraquat poisoning mortality rates ranging from 33.0% to 91.7% primarily due to multiple organ failure and pulmonary fibrosis [8]. Paraquat poisoning can manifest as acute and chronic toxicity. The major acute effects can result in both local and systemic manifestations. Local effects include ulceration of the skin, lips, tongue, pharynx, and esophagus. Systemic effects involve multiple organ failure, including liver insufficiency, acute kidney injury, respiratory failure, and convulsions [3, 5, 8]. The severity of paraquat poisoning is classified into three categories: mild, moderate-to-severe, and fulminant. Mild poisoning is characterized by minor gastrointestinal tract disorders. Moderate-to-severe poisoning often leads to acute renal failure, acute hepatitis, acute lung injury, and progressive pulmonary fibrosis. Fulminant poisoning results in multiple organ failure and death within a few days [9, 10].

The high mortality rates associated with paraquat poisoning can be attributed to the lack of effective treatment. Currently, there is no specific antidote or universally accepted treatment guidelines for paraquat intoxication [3, 5, 8]. Medical management options range from supportive care, including gastrointestinal decontamination within 2–4 h of ingestion, to various combinations of immunosuppressive therapy (such as dexamethasone and cyclophosphamide), antioxidants (such as vitamin C, vitamin E, and *N*-acetyl cysteine), and hemoperfusion within 2 h of ingestion [3, 5].

In Thailand, a few studies have been published on acute paraquat poisoning. In a previous study, the severity of acute paraquat poisoning was evaluated in eight autopsy cases. The survival periods ranged from 26 h to 59 days. The main causes of death were circulatory collapse, acute alveolar injury, acute tubular necrosis, hepatic necrosis, and cerebral edema [11]. Another case report involving a Thai male farmer demonstrated that dermal exposure to a paraquat solution resulted in serious systemic toxicity, including renal failure, respiratory failure, and hepatic damage [12]. In a separate study, factors associated with the chance of survival in patients with paraquat poisoning were analyzed. The study identified age, amount of paraquat ingested, and white blood cell count at admission as factors positively correlated with mortality [13]. In support of these previous data, our study focused on the in-hospital fatality rate, presumed causes, clinical presentation, outcomes, and management of paraquat intoxication in healthcare facilities across Thailand. Therefore, the primary objective aimed to identify the factors associated to mortality in patients who visited a tertiary referral hospital in Thailand and were poisoned by paraquat. The secondary objective was to assess the clinical presentation and outcomes of all individuals exposed to paraquat.

## Methods

### Study design

The present study was a cross-sectional retrospective review conducted at a tertiary care hospital over five years. The primary objective aimed to identify the factors associated with death in patients poisoned by paraquat. The secondary objective was to assess the clinical presentation and outcomes of all individuals exposed to paraquat.

### Study protocol

The study protocol was approved by the institutional review board of the hospital. The characteristics of patients, causes, clinical presentation, outcomes, and management of paraquat intoxication were gathered from the medical charts and recorded for evaluation by physicians. The hospital information system, including the Electronic Health Record (EHR), was utilized to identify patients with acute paraquat toxicity and retrieve medical data, laboratory measurements, and prescription records for review.

The amount of paraquat ingested was quantified as follows: “a small amount or a teaspoon” was considered as 5 ml, “a mouthful” as 25 ml, “a small cup” as 100 ml, “a glass” as 300 ml, and “a bottle” as 500 ml [14, 15]. The severity of the poisoning was defined based on clinical characteristics and outcomes. Patients with mild poisoning either exhibited no symptoms or experienced mild gastrointestinal tract disorders. All of these patients fully recovered. Patients with moderate to severe poisoning presented with non-specific symptoms such as local gastrointestinal symptoms, renal failure, hepatic dysfunction, and pulmonary fibrosis, which could manifest for several weeks. Most of these patients experienced death, which might be delayed for 2–3 weeks. Patients with fulminant poisoning experienced multiple organ failures, including cardiac, respiratory, hepatic, renal, and neurological failure. All of these patients died, typically within hours, without delays exceeding a week [16]. The medical management options varied depending on the clinical presentations. Treatment approaches included gastrointestinal decontamination, immunosuppressive therapy, antioxidants, and, in some cases, hemoperfusion.

Patients were divided into two groups based on their outcomes: survivors and non-survivors. Survivors were defined as patients who either recovered or showed improvement in clinical outcomes. Non-survivors were defined as patients who did not demonstrate improvement in clinical outcomes or died. The factors that might be associated with clinical outcomes were compared between the two groups.

### Study setting and population

The study population comprised patients who were admitted to a tertiary referral hospital in Thailand with acute paraquat poisoning. Eligible patients presented with acute paraquat toxicity between 1 January 2016 and 31 December 2020, covering 5 years. No specific sample size was calculated for the study due to the rarity of paraquat poisoning. All patients who met the inclusion criteria and were identified during the study period were considered eligible. The inclusion criteria encompassed all patients who presented to the Emergency Department of the tertiary referral hospital with confirmed paraquat poisoning, which was established through a history of paraquat exposure and a positive urine sodium dithionite test result. Additionally, patients with complete medical records documenting their clinical history, treatment, and outcomes related to paraquat poisoning were included. The exclusion criteria applied to individuals with suspected paraquat poisoning but without confirmation through a urine sodium dithionite test. Patients with incomplete medical records necessary for a comprehensive assessment of their condition were also excluded, as well as those with severe coexisting medical conditions or comorbidities that could potentially confound the study’s outcomes such as chronic kidney and liver diseases [17].

### Statistical analysis

Descriptive statistics were employed to calculate the baseline characteristics of the patients, presumed causes, clinical presentation, outcomes, and management. The data were presented as the number (%) of patients and the mean ± standard deviations (SD). Factors that were potentially associated with death were analyzed using the t-test for continuous variables, the Chi-square test, or Fisher’s exact test. Significant variables were further examined using multivariate logistic regression to predict mortality. The analyses were conducted using SPSS software version 28.0 (SPSS Inc., Chicago, IL, USA), and p-values less than 0.05 were considered statistically significant.

### Ethics statement

This study was approved by the Human Experimentation Committee at Nakornping Hospital, Chiang Mai 50180, Thailand. The ethics approval reference number is 113/63. Patient consent was not required because this study was retrospective, involving the review of a pre-existing confidential database from the hospital. The results of this study are reported anonymously.

## Results

The medical records of 148 patients were examined during the study period. The majority of the patients were male (75.7%). The average age of the patients was 37 ± 15 years, ranging from 1 to 71 years. The mean length of hospital stay was 4 ± 3 days, ranging from 1 to 19 days. All of the patients resided in rural areas (100.0%). Among the patients with paraquat poisoning, comorbid conditions were present in 52.0% of cases, a history of drug addiction in 19.2%, psychiatric problems in 71.8%, and a history of previous attempted suicide in 14.3% (Additional file 1: Table S1). The most prevalent causes of poison exposure were intentional self-poisoning (87.1%), accidental poisoning (12.1%), and occupational poisoning (0.7%), primarily through oral ingestion (98.6%). The mean quantity of paraquat ingested was 120.5 ± 210.6 ml, ranging from 1 to 1250 ml. The average time interval from poison exposure to hospital arrival was 17.9 ± 31.1 h, ranging from 0.5 to 168 h (Additional file 2: Table S2).

The in-hospital fatality rate was 21.8%. However, the majority of patients showed improvement in clinical outcomes (44.4%), followed by patients who did not show improvement (33.1%). The most common degrees of severity were moderate-to-severe (73.4%), followed by fulminant (18.8%), and mild (7.8%). Medical management included gastric lavage, administration of activated charcoal, and use of fuller’s earth, which were performed for 55.4%, 60.8%, and 5.4% of patients, respectively. Other treatments included immunosuppressive therapy, specifically cyclophosphamide for 92.6% of patients, and dexamethasone for 95.9% of patients. Antioxidants, such as vitamin C, vitamin E, and *N*-acetyl cysteine, were administered to 86.5%, 87.8%, and 17.5% of patients, respectively. Additionally, hemoperfusion was performed for 18.9% of patients (Additional file 2: Table S2). The clinical presentations of paraquat toxicity were as follows: gastrointestinal tract disorder (82.3%), renal disorder (71.7%), pulmonary disorder (45.3%), hepatic disorder (36.2%), cardiovascular system (CVS) disorder (29.0%), central nervous system (CNS) disorder (14.5%), and dermatological disorder (10.1%). The mean number of organ failures was 3 ± 1, ranging from 2 to 4 (Table 1).

**Table 1:** Clinical Presentation of Paraquat Intoxication

Characteristics	Number of patients (%)
Gastrointestinal tract disorder (n = 141)	
- Yes^a,b^	116 (82.3)
- Nausea, vomiting, diarrhea	89 (28.6)
- Esophageal ulceration, dysphagia, heartburn	87 (28.0)
- Oral ulceration, white patch (paraquat tongue)	67 (21.6)
- Others	68 (21.8)
Renal disorder (n = 138)	
- Yes^a,c^	99 (71.7)
- Acute kidney injury/acute renal failure	74 (43.3)
- Hypokalemia	46 (26.9)
- Lactic acidosis/metabolic acidosis	25 (14.6)
- Others	26 (15.2)
Pulmonary disorder (n = 139)	
- Yes^a,d^	63 (45.3)
- Tachypnea, dyspnea	69 (39.7)
- Acute respiratory failure	28 (16.1)
- Lung infiltration	11 (6.3)
- Others	66 (37.9)
Hepatic disorder (n = 138)	
- Yes^a,e^	50 (36.2)
- Elevated liver enzymes	59 (66.3)
- Liver failure	11 (12.4)
- Acute hepatitis	8 (8.9)
- Others	11 (12.4)
Cardiovascular system disorder (n = 138)	
- Yes^a,f^	40 (29.0)
- Tachycardia	25 (39.7)
- Shock	15 (23.8)
- Others	23 (36.5)
Central nervous system disorder (n = 138)	
- Yes^a,g^	20 (14.5)
- Loss of consciousness	20 (74.1)
- Others	7 (25.9)
Dermatological disorder (n = 138)	
- Yes	14 (10.1)
- Sweating	6 (42.8)
- Conjunctivitis	4 (28.6)
- Others	4 (28.6)

^a^More than one clinical presentation might be found in one patient

^b^n = 311

^c^n = 171

^d^n = 174

^e^n = 89

^f^n = 63

^g^n = 27

There were significant differences between the two groups (survivors and non-survivors) in terms of the reasons for exposure to poison, the amount of paraquat ingested, and clinical presentations including renal disorder, pulmonary disorder, hepatic disorder, CVS disorder, CNS disorder, multiorgan failure, and degree of severity (Table 2). Non-survivors had a significantly higher number of cases of intentional self-poisoning (50.4%) compared to survivors (37.0%) (*P* = 0.017). The mean amount of paraquat ingested by non-survivors was ten times higher than that of survivors (206.7 ± 257.8 ml vs. 20.6 ± 42.1 ml, *P* < 0.000). Renal disorder, pulmonary disorder, hepatic disorder, CVS disorder, CNS disorder, and multiorgan failure were significantly more frequent in non-survivors (*P* < 0.000). All patients with fulminant poisoning were in the non-survivors group, while all patients with mild poisoning were in the survivors group (*P* < 0.000). There were no significant differences between survivors and non-survivors in terms of medical management (Table 2). In the multivariate analysis, only the amount of paraquat ingested (odds ratio [OR] = 25.04; 95% confidence interval [CI] = 4.12–152.03), as well as the presence of pulmonary disorder (OR = 24.43; 95% CI = 3.73–160.02) and cardiovascular system disorder (OR = 13.02; 95% CI = 1.51–112.14), were found to be significantly associated with death (Table 3).

**Table 2:** Factors associated with non-surviving paraquat-poisoned patients

Characteristics	Number of patients (%)		*P*-value
	Survivors	Non-survivors	
Mean age (years ± SD) (n = 133)	36 ± 15	38 ± 16	0.441
Reason for exposure to poison (n = 127)			
- Intentional	47 (37.0)	64 (50.4)	0.017*
- Accidental	12 (9.5)	4 (3.1)	
Mean amount of paraquat ingested (ml ± SD) (n = 106)	20.6 ± 42.1	206.7 ± 257.8	0.000*
Period from poison exposure to arrival at hospital (h ± SD) (n = 111)	24.0 ± 34.4	13.8 ± 28.6	0.090
Gastrointestinal tract disorder (n = 129)			
- No	11 (8.5)	7 (5.4)	0.127
- Yes	45 (34.9)	66 (51.2)	
Renal disorder (n = 126)			
- No	28 (22.2)	4 (3.2)	0.000*
- Yes	27 (21.4)	67 (53.2)	
Pulmonary disorder (n = 127)			
- No	51 (40.2)	17 (13.4)	0.000*
- Yes	4 (3.1)	55 (43.3)	
Hepatic disorder (n = 126)			
- No	42 (33.3)	37 (29.4)	0.006*
- Yes	13 (10.3)	34 (27.0)	
Cardiovascular system disorder (n = 126)			
- No	52 (41.2)	36 (28.6)	0.000*
- Yes	3 (2.4)	35 (27.8)	
Central nervous system disorder (n = 126)			
- No	55 (43.6)	52 (41.3)	0.000*
- Yes	0 (0.0)	19 (15.1)	
Dermatological disorder (n = 126)			
- No	49 (38.9)	64 (50.8)	1.000
- Yes	6 (4.8)	7 (5.5)	
Multiorgan failure (n = 72)			
- No	28 (38.9)	10 (13.9)	0.000*
- Yes	0 (0.0)	34 (47.2)	
Degree of severity (n = 126)			
- Mild	10 (8.0)	0 (0.0)	0.000*
- Moderate-to-severe	45 (35.7)	47 (37.3)	
- Fulminant	0 (0.0)	24 (19.0)	
Treatment for paraquat intoxication (n = 148)			
- Gastric lavage	31 (23.3)	46 (34.6)	0.218
- Activated charcoal	28 (21.1)	53 (39.8)	0.003*
- Hemoperfusion	10 (7.5)	17 (12.8)	0.392
- Cyclophosphamide	55 (41.4)	67 (50.4)	1.000
- Dexamethasone	57 (42.9)	70 (52.6)	1.000
- Vitamin C	54 (40.6)	61 (45.9)	0.318
- Vitamin E	53 (39.8)	64 (48.1)	1.000

^*^Statistical significance

**Table 3:** Multivariate Logistic Regression Analysis of Variables Associated With Death

Variables	Univariate			Multivariate		
	OR	95% CI	*p*-value	OR	95% CI	*p*-value
Reason for exposure to poison (Intentional vs. accidental)	4.09	1.24–13.46	0.021	**–**	**–**	**–**
Amount of paraquat ingested (ml) (> 15 vs. ≤ 15)	22.45	8.07–62.44	0.000	25.04	4.12–152.03	0.000
Renal disorder (Yes vs. No)	17.37	5.56–54.26	< 0.001	**–**	**–**	**–**
Pulmonary disorder (Yes vs. No)	41.25	13.01–130.79	< 0.001	24.43	3.73–160.02	0.001
Hepatic disorder (Yes vs. No)	2.97	1.37–6.46	0.006	**–**	**–**	**–**
Cardiovascular system disorder (Yes vs. No)	16.85	4.81–59.02	< 0.001	13.02	1.51–112.14	0.019

*OR* odds ratios, *CI* confidence interval

The reference category is survived, paraquat-poisoned patients

## Discussion

In many developing countries, including Thailand, acute paraquat poisoning is a significant health concern due to its high mortality rate [8, 9]. This study, which analyzed 148 cases over five years, is one of the largest studies conducted on paraquat poisoning in Thailand to date. The in-hospital fatality rate observed in this study was 21.8%. Additionally, 33.1% of patients did not show improvement in their clinical outcomes. Previous studies have reported in-hospital fatality rates ranging from 46.3% to 55.2% [10, 18]. Similarly, high mortality rates ranging from 33.0% to 91.7% have been observed in previous studies on acute paraquat poisoning [8, 9]. The current study revealed that 73.4% of patients had moderate-to-severe poisoning, while 18.8% of patients had fulminant poisoning. Many previous studies have classified paraquat poisoning into three categories based on the amount of paraquat ingested [9, 10]. However, determining the exact volume of paraquat ingested was not possible in our study. We could only approximate the amount of paraquat based on the information provided by the patients or their relatives. Our study assessed the severity of paraquat poisoning based on clinical manifestations and outcomes. It is important to note that no specific antidote or effective treatment has been identified to reduce mortality in cases of paraquat intoxication [3, 5, 10]. Our findings revealed no significant differences in medical management between survivors and non-survivors. These results are consistent with previous studies that have shown weak evidence for the effectiveness of hemoperfusion, immunosuppression, and antioxidants [3, 10, 19, 20]. A multicenter retrospective study also found no association between hemoperfusion and increased 60-day survival in patients with acute paraquat poisoning [21]. However, some studies have indicated that early hemoperfusion within 4 h of ingestion may improve survival rates and clinical outcomes in severe cases of paraquat poisoning [22, 23]. In this study, 81.1% of patients were unable to afford hemoperfusion therapy due to financial constraints. A previous meta-analysis study suggested that immunosuppressive therapy may reduce mortality in patients with moderate to severe poisoning, but further studies are needed to confirm this finding [24]. Given paraquat’s role as an oxidative stress inducer, several studies have proposed antioxidant therapy as a potential treatment [2, 25]. One clinical study demonstrated that high-dose, long-term antioxidant therapy significantly improved survival rates as well as lung and liver function [26]. However, additional clinical studies are required to validate the efficacy and safety of antioxidant therapy [26, 27]. A potent emetic was added to paraquat formulations to prevent paraquat absorption [10]. Thus, this study revealed that the most common clinical presentations of paraquat toxicity were gastrointestinal tract disorders such as nausea, vomiting, and diarrhea. Renal and pulmonary disorders were also observed in paraquat-poisoned patients, as paraquat is distributed to all organs, particularly the kidneys, and lungs [3, 6, 7].

Our results showed that non-survivors had a significantly higher number of intentional self-poisoning cases (50.4%) compared to survivors (37.0%) (*P* = 0.017). The mean amount of paraquat ingested by non-survivors was ten times higher than that of survivors (206.7 ± 257.8 ml vs. 20.6 ± 42.1 ml, *P* < 0.000). Furthermore, multivariate analysis revealed that the amount of paraquat ingested was associated with death (OR = 25.04; 95% CI = 4.12–152.03). This finding is consistent with a previous study, which found that suicidal poisoning tends to be more severe due to the consumption of higher doses of paraquat [9]. Another previous study also emphasized the importance of the ingested amount of paraquat as a prognostic factor for patients. It is essential to administer activated charcoal to reduce the absorption of paraquat [28]. However, the effectiveness of activated charcoal in paraquat poisoning has not been reported in the literature [23]. Moreover, there is evidence suggesting that routine administration of single and multiple doses of activated charcoal does not provide any benefit in improving clinical outcomes or reducing mortality rates [29, 30]. Our findings are consistent with this, indicating that the administration of activated charcoal does not offer significant help in managing paraquat poisoning. Further studies with a larger sample size may be useful to investigate this further.

Published studies have identified several parameters that can be used to predict the mortality rates of paraquat-poisoned patients. These studies have found that certain factors, including the number of white blood cells, blood sugar levels, serum creatinine levels, and liver enzymes (aspartate aminotransferase, alanine aminotransferase), play a significant role in predicting the severity of acute paraquat poisoning [9, 31]. Furthermore, previous research has indicated that certain clinical presentations, such as the presence of systemic inflammatory response syndrome, early tachycardia, and renal failure, can be used to predict early mortality [8, 31]. Interestingly, our study revealed a significant association between non-survivors and the presence of renal disorder, pulmonary disorder, hepatic disorder, CVS disorder, CNS disorder, and multiorgan failure (*P* < 0.000). Additionally, all patients with fulminant poisoning were classified as non-survivors, while all patients with mild poisoning belonged to the survivors’ group (*P* < 0.000). Furthermore, our multivariate analysis indicated that clinical presentations, particularly pulmonary (OR = 24.43; 95% CI = 3.73–160.02) and cardiovascular system disorders (OR = 13.02; 95% CI = 1.51–112.14), were significantly associated with death. Therefore, the presence of pulmonary and cardiovascular system disorders holds the potential as valuable predictors of mortality rates.

### Study limitations

In this present study, it is important to acknowledge that the data were collected from a single tertiary referral hospital, which may limit the generalizability of the results. Furthermore, as the study was retrospective, the availability and quality of clinical records could have impacted the accuracy of the findings. Certain data, such as the number of deaths occurring after discharge, were not able to be collected. Additionally, the amount of paraquat consumed was reliant on the history provided by the patients or their relatives. To enhance the strength of future research, it would be beneficial to conduct prospective and multicenter studies. The inclusion of serum paraquat concentration or novel biomarkers may also prove valuable in improving the predictive accuracy of outcomes. Importantly, the potential for selection bias represents a significant limitation. Due to the rarity of paraquat poisoning, a specific sample size was not calculated. Instead, this study included all eligible patients based on predefined criteria. These clearly defined inclusion and exclusion criteria were employed to mitigate this limitation.

## Conclusions

In conclusion, acute paraquat poisoning remains a significant health issue in Thailand. This study revealed that non-survivors had a significantly higher incidence of intentional self-poisoning (*P* = 0.017). The amount of paraquat ingested was found to be ten times higher in non-survivors compared to survivors (206.7 ± 257.8 ml vs. 20.6 ± 42.1 ml, *P* < 0.000). Additionally, the study identified the ingested amount of paraquat, as well as pulmonary and cardiovascular system disorders, as prognostic factors for mortality rates. These findings provide valuable insights for physicians to predict the prognosis and mortality of paraquat poisoning. Furthermore, this study raises concerns about the acute toxic effects of paraquat and emphasizes the importance of public education and awareness among healthcare professionals regarding the toxic consequences of paraquat poisoning, to prevent pesticide misuse and suicide attempts. It is worth noting that there were no significant differences in medical management between survivors and non-survivors, underscoring the urgent need for the development of novel treatments.

### Supplementary Information

**Additional file 1: Table S1.** Demographic characteristics of the paraquat-intoxicated patients.

**Additional file 2: Table S2.** Presumed causes, clinical presentation, clinical outcomes, and treatment for paraquat intoxication.

## Data Availability

All data generated or analyzed during this study, including supplementary tables, are included in this published article. Additionally, the datasets used in the current study are available from the corresponding author upon reasonable request.
