# Supplementary material for: Predicting mortality in paraquat poisoning through clinical findings, with a focus on pulmonary and cardiovascular system disorders
Source: J Pharm Policy Pract. 2023 Oct 20;16:123. doi: 10.1186/s40545-023-00635-z (PMC10588157; doi:10.1186/s40545-023-00635-z)
Supplement: Supplementary file 1 — Additional file 1: Table S1. Demographic characteristics of the paraquat-intoxicated patients. [file 40545_2023_635_MOESM1_ESM.docx]

**Table S1.** Demographic characteristics of the paraquat-intoxicated patients

| **Characteristics** | **Number of patients (%)** |
| --- | --- |
| Gender (n=148)   - Male - Female | 112 (75.5)  36 (24.3) |
| Mean age (years ± SD) (n=148) | 37±15 |
| Mean length of hospital stay (days ± SD) (n=147) | 4±3 |
| Season of admission (n=148)   - Summer - Rainy - Winter | 29 (19.6)  73 (49.3)  46 (31.1) |
| Living area (n=125)   - Rural - Urban | 125 (100)  0 (0) |
| Comorbid condition (n=123)   - No - Yes^a,b^ - Hypertension - Diabetes mellitus - Others | 59 (48.0)  64 (52.0)  9 (23.0)  4 (10.3)  26 (66.7) |
| Drug addiction (n=78)   - No - Yes^c,d^ - Amphetamines - Morphine - Others | 63 (80.8)  15 (19.2)  14 (73.6)  2 (10.5)  3 (15.9) |
| Psychiatric problem (n=110)   - No - Yes^e,f^ - Major depressive disorder (MDD) - Adjustment disorder - Psychotic disorder | 31 (28.2)  79 (71.8)  38 (35.5)  34 (31.8)  35 (32.7) |
| History of suicide attempts (n=63)   - No - Yes | 54 (85.7)  9 (14.3) |
| Co-ingestion with other drugs (n=72)   - No - Yes^g,h^ - Alcohol - Glyphosate - Methomyl - Others | 32 (44.4)  40 (55.6)  32 (65.3)  2 (4.1)  2 (4.1)  13 (26.5) |

^a^n=39, ^b^More than 1 comorbid condition might be found in 1 patient.

^c^n=19, ^d^More than 1 drug addiction might be found in 1 patient.

^e^n=107, ^f^More than 1 psychiatric problem might be found in 1 patient.

^g^n=49, ^h^More than 1 co-ingestion might be found in 1 patient.
