# Supplementary material for: Predicting mortality in paraquat poisoning through clinical findings, with a focus on pulmonary and cardiovascular system disorders
Source: J Pharm Policy Pract. 2023 Oct 20;16:123. doi: 10.1186/s40545-023-00635-z (PMC10588157; doi:10.1186/s40545-023-00635-z)
Supplement: Supplementary file 2 — Additional file 2: Table S2. Presumed causes, clinical presentation, clinical outcomes, and treatment for paraquat intoxication. [file 40545_2023_635_MOESM2_ESM.docx]

**Table S2.** Presumed causes, clinical presentation, clinical outcomes, and treatment for paraquat intoxication

| **Characteristics** | **Number of patients (%)** |
| --- | --- |
| Reason for exposure to poison (n=140)   - Intentional - Accidental - Occupational | 122 (87.1)  17 (12.1)  1 (0.7) |
| Route of exposure (n=144)   - Oral ingestion - Dermal contact - Inhalation | 142 (98.6)  1 (0.7)  1 (0.7) |
| Mean amount of paraquat ingested (ml ± SD) (n=110) | 120.5±210.6 |
| Period from poison exposure to arrival at hospital  (h ± SD) (n=114) | 17.9±31.1 |
| Clinical presentation   - Gastrointestinal tract disorder (n=141) - Renal disorder (n=138) - Pulmonary disorder (n=139) - Hepatic disorder (n=138) - Cardiovascular system disorder (n=138) - Central nervous system disorder (n=138) - Dermatological disorder (n=138) | 116 (82.3)  99 (71.7)  63 (45.3)  50 (36.2)  40 (29.0)  20 (14.5)  14 (10.1) |
| Multiorgan failure (n=73) | 34 (46.6) |
| Mean number of organ failure (number ± SD) (n=34) | 3±1 |
| Clinical outcomes (n=133)   - Recovered - Improved - Not improved - Dead | 1 (0.7)  59 (44.4)  44 (33.1)  29 (21.8) |
| Mean time from onset to death (days ± SD) (n=28) | 2±4 |
| Degree of severity (n=128)   - Mild - Moderate-to-severe - Fulminant | 10 (7.8)  94 (73.4)  24 (18.8) |
| Treatment for paraquat intoxication (n=148)   - Gastric lavage - Activated charcoal - Hemoperfusion - Cyclophosphamide - Dexamethasone - Vitamin C - Vitamin E - N-acetylcysteine (n=114) | 82 (55.4)  90 (60.8)  28 (18.9)  137 (92.6)  142 (95.9)  128 (86.5)  130 (87.8)  20 (17.5) |
